# A case of fitness to work in a worker with COPD using the exercise stress test 

**DOI:** 10.1186/s40557-015-0074-z

**Published:** 2015-12-11

**Authors:** Yewon Kim, Kyungyong Jung, Ji Young Ryu, Dae Hwan Kim, Sangyoon Lee

**Affiliations:** Department of Occupational and Environmental Medicine, Inje University Haeundae Paik Hospital, 875 Haeundae-ro, 612-862 Busan, Haeundae-Gu South Korea

**Keywords:** Chronic obstructive pulmonary disease, Fitness to work, Exercise stress test

## Abstract

**Background:**

Chronic obstructive pulmonary disease (COPD) is a chronic respiratory disease characterized by persistent airflow limitation. Therefore, both work ability and workday length may be affected in individuals with this disease. We studied a worker with suspected COPD and assessed fitness to work using post-bronchodilator spirometry, symptom assessment scales, and the exercise stress test.

**Case report:**

The patient was a 58-year-old man due to work as a field supervisor in the ship construction sector. He had a 40 pack-year smoking history and experienced occasional dyspnea when climbing stairs. He visited this hospital to receive cardiopulmonary function tests and to determine his ability to work. Post-bronchodilator spirometry revealed severe irreversible airway obstruction corresponding to a modified Medical Research Council grade of 2 on the dyspnea scale. His COPD Assessment Test score was 12, placing him in patient group D (high risk, more symptoms) based on the Global Initiative for Chronic Obstructive Lung Disease classification system. His maximum oxygen uptake (VO_2max_) was determined to be 19.16 ml/kg/min, as measured by the exercise stress test, and his acceptable workload for 8 h of physical work was calculated to be 6.51 ml/kg/min. His work tasks required an oxygen demand of 6.89 ml/kg/min, which exceeded the acceptable workload calculated. Accordingly, he was advised to adjust the work tasks that were deemed inappropriate for his exercise capacity.

**Conclusion:**

As COPD incidence is expected to rise, early COPD diagnosis and determination of fitness to work is becoming increasingly important. Performing the exercise stress test, to evaluate the functional capacity of workers with COPD, is considered an acceptable solution.

## Background 

According to the Korean National Health and Nutrition Examination Survey of 2008, 13.4 % of the population > 40 years of age was affected by COPD: the prevalence of COPD in males was 19.4 % compared to 7.9 % in females [1]. Smoking is known to be the most significant cause of COPD, but there is increasing evidence pointing toward important contributions from occupational exposures, including organic and inorganic dusts, gases, and fumes [2]. A recent study in the US demonstrated that the cause of COPD in 19.2 % of patients was associated with work and workplaces [3].

As exercise capacity decreases, COPD patients may experience lower work capability resulting in shorter workdays. Additionally, exposure to the pollutants exhausted in workplaces can aggravate the symptoms of COPD, highlighting the importance of examining the workplace environment for contributing risk factors. Thus, monitoring workplace environments and evaluating disease progress and functional capability are important to ensure the protection of workers, as is prescribing workers with appropriate tasks relevant to their physical health status [4].

The severity of COPD and necessity for medications are determined based on the standards established by the Global Initiative for Chronic Obstructive Lung Disease (GOLD). However, it is difficult to assess work ability objectively since there are no explicit regulations or guidelines. For example, the degree of dyspnea needed to hinder work ability is subjective and cannot be used as a basis for evaluating capacity to work.

Therefore, it is essential to establish criteria that can be used to evaluate different aspects of physical ability that correlate well with capacity to work. Indeed, objective standards should be used to determine the degree of work ability required for certain tasks, as well as the work intensity and number of work hours that workers can undertake without exacerbating the disease. Since the exercise stress test is useful for evaluating exercise capacity decline, we used it to measure cardiopulmonary function and evaluate fitness to work in a patient with suspected COPD [5, 6].

Accordingly, this study suggests assessing fitness to work in individuals with COPD by evaluating disease severity , work tasks, and work environment, and by performing an exercise stress test.

## Case presentation

**Fig. 1:**
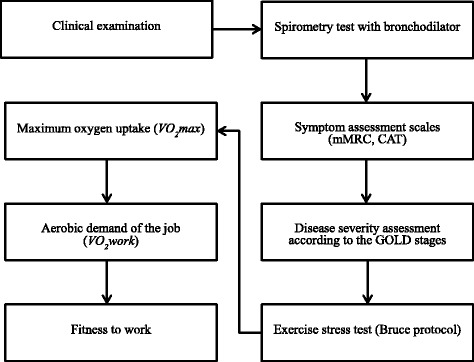
Flow chart for fitness to work in workers suspected of having COPD. mMRC, modified Medical Research Council; CAT, COPD Assessment Test; GOLD, Global Initiative for Chronic Obstructive Lung Disease

The patient studied was a 58-year-old man who had been working as a shipbuilding supervisor in an office setting performing primarily sedentary jobs. However, he was expected to start working on a construction site for ships used to transport gas and oil, which involved 3 h of field supervising per 8-h workday. His field tasks involved climbing stairs, walking, and moving in small, enclosed spaces. He was a smoker and complained of dyspnea when walking or climbing. He visited the Department of Occupational and Environmental Medicine to examine his capacity to work and perform scheduled tasks without negative health consequences, with respect to cardiopulmonary function. Figure 1 depicts the steps taken to test the patient’s fitness to work. This study was approved by the Institutional Review Board of Haeundae Paik Hospital.

Ship construction site workers may be exposed to iron oxide dust, heavy metals, and harmful gases. Following a work environment evaluation performed in the same workplace in the latter half of 2013, we calculated the amount of harmful inhalable substances the patient could be exposed to in the process of welding and grinding. The arithmetic mean exposure to welding fumes and iron oxide dust were 2.73 (±1.35) and 2.94 (±1.04) mg/m^3^ respectively, which does not exceed the acceptable limit in Korea. The welding fumes included iron oxide, titanium dioxide, manganese and manganese compounds, and aluminum and aluminum compounds. Grinding generated various types of dust.

The patient’s baseline physical characteristics and lifestyle traits were as follows: height, 179 cm; body weight, 132 kg; waist circumference, 129 cm; body mass index, 41.1 kg/m^2^; blood pressure, 160/90 mmHg; pulse, 89 beats/min; respiratory rate, 23 breaths/min; body temperature, 36.8 °C; 40 pack-year smoking history; and consumption of 10–12 units of alcohol per week. Stethoscope examination indicated mild wheezing. He had been medicated for hypertension and dyslipidemia for the past 2 years.

Although the patient was not affected by a chronic cough or sputum production, he was suspected of having COPD because of his 40 pack-year smoking history, mild wheezing, and dyspnea during exercise. To rule out other conditions with similar symptoms, such as asthma, his pre-bronchodilator spirometry results (FEV_1_/FVC, 0.46; FEV_1_, 1.81 L, 47 % predicted; FVC, 3.90 L, 82 % predicted) were compared with his post-bronchodilator spirometry results (FEV_1_/FVC, 0.46; FEV_1_, 1.89 L, 49 % predicted; FVC, 4.09 L, 86 % predicted). Post-bronchodilator spirometry was performed 15 min after the inhalation of 400 μg bronchodilator salbutamol. The patient was diagnosed with COPD as the improvements in both FEV_1_ and FVC were less than the predicted 12 %, the absolute increase was below 0.2 L, and the FEV_1_/FVC ratio was < 0.7. These results indicate that the patient’s airflow limitations correspond to a GOLD 3 rating (severe, FEV_1_/FVC < 0.7, 30 % ≤ FEV_1_ < 50 % predicted).

**Fig. 2:**
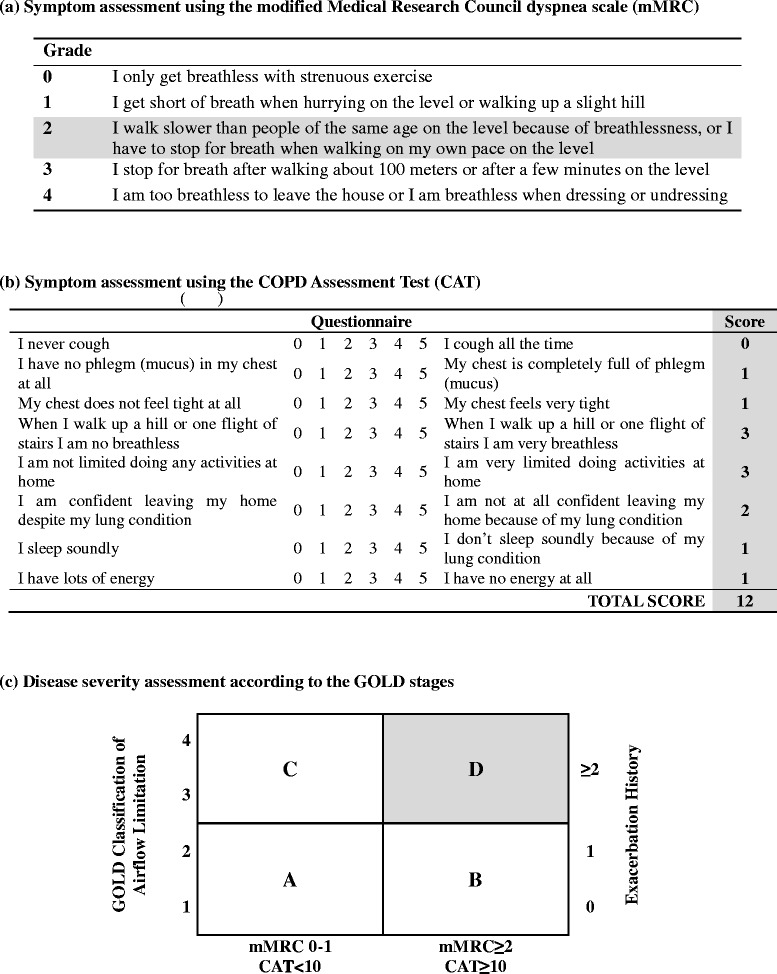
COPD assessment using symptoms, breathlessness, spirometric classification, and risk of exacerbations. The patient had a GOLD airflow limitation rating of 3, dyspnea of mMRC grade 2, and a CAT score of 12; therefore, he was assigned to patient group D (high risk, more symptoms)

To evaluate the severity of COPD, the patient’s degree of dyspnea was assessed using the modified Medical Research Council (mMRC) dyspnea scale, and his symptoms were assessed using the COPD Assessment Test (CAT). He had no history of acute COPD exacerbation, but experienced shortness of breath and flagging pace when walking on level ground, resulting in a mMRC grade of 2. He experienced shortness of breath when climbing slopes and stairs and also complained of chest pain that limited normal life at home, resulting in a CAT score of 12. High-risk individuals are characterized by a history of acute exacerbations twice per year or an exacerbation leading to hospital admission once per year. Regardless of exacerbation history, patients are categorized into the high-risk group if they are affected by an GOLD airflow limitation score of 3 or 4 according to the pulmonary function test. High-risk individuals with CAT scores > 10, or mMRC grades > 2, are classified as belonging to patient group D [7]. Since the patient had a GOLD airflow limitation rating of 3, a mMRC dyspnea grade of 2, and a score of 12 on the CAT, he was classified into patient group D (Fig. 2).

Because FEV_1_ exceeded 40 % and the patient did not show signs of ventilatory failure, arterial blood gas analysis was not performed. Moreover, the alpha1-antitrypsin deficiency-screening test was not conducted because the patient was > 45 years of age and there was no family history of this disorder. Chest X-rays revealed no specific findings and we did not detect polycythemia, which can occur in relation to hypoxemia.

The exercise stress test was performed to evaluate exercise capacity and to discover possible abnormalities during exercise. The test was stopped at Stage 4 (6.7 km/h) because the patient showed abnormal elevation of systolic blood pressure (210/80 mmHg) and complained of dyspnea. He did not experience chest pain and his electrocardiography did not show ST segment depression. The maximum oxygen uptake (VO_2max_) was calculated to be 19.16 ml/kg/min (total time completed = 5 min 38 s), which was classified as ‘very poor’ for his age group [8].

The patient’s tasks at work included the following physical activities and durations: sedentary work, 300 min; car driving, 30 min; walking slowly, 60 min; walking normally, 20 min; standing and speaking, 30 min; ascending stairs, 20 min; and descending stairs, 20 min. Ainsworth et al. provided aerobic demands (VO_2work_) for each of the following tasks: sedentary work, 5.3 ml/kg/min; car driving, 7.0 ml/kg/min; walking slowly, 7.0 ml/kg/min; walking normally, 12.3 ml/kg/min; standing and speaking, 10.5 ml/kg/min; ascending stairs, 14.0 ml/kg/min; and descending stairs, 12.25 ml/kg/min. The mean aerobic demand for the patient’s activities was 6.89 ml/kg/min (Table 1) [9]. Wu and Wang suggested that 34 % of an individual’s VO_2max_ is an acceptable workload for a general 8-h physical workday, such that 6.51 ml/kg/min (34 % of the VO_2max_ of 19.16 ml/kg/min) was recommended as the patient’s workload [10]. However, the expected tasks required 6.89 ml/kg/min, which exceeded the 6.51 ml/kg/min cut-off, and thus these tasks were found to be unsuitable for the patient.

**Table 1:** Investigation of energy requirements of the job

	VO_2work_(ml/kg/min)^*^	Work time (min)	VO_2work_ × time (ml/kg)
Sedentary work	5.3	300	1,590
Car driving	7.0	30	210
Walking slowly	7.0	60	420
Walking normally	12.3	20	246
Standing and speaking	10.5	30	315
Ascending stairs	14.0	20	280
Descending stairs	12.3	20	246
Total		480	3307

Mean aerobic demand for these activities = 6.89 ml/kg/min (3,307/480). VO_2work,_ aerobic demand of the job

^*^VO_2work_ values taken from Ainsworth et al. [9]

Since the patient was in patient group D (high risk, more symptoms) and required medication for COPD, it was necessary to limit his tasks to sedentary work. COPD exacerbation could endanger the patient and his colleagues at work; therefore, health personnel at his place of employment recognized that continuing certain tasks could impose considerable physical burden on the patient. Moreover, smoking cessation and wearing a powered air purifying respirator (PARP) when exposed to environmental factors that could aggravate the disease were essential to the patient’s health.

## Conclusions

We performed following medical interventions to assess fitness to work for a worker with COPD in this case. Post-bronchodilator spirometry was utilized to diagnose COPD and COPD severity was assessed based on GOLD classification. Exercise stress test, calculating maximum oxygen uptake, and analyzing work ability of the worker were performed. Because the work environment and tasks could aggravate diseases of the worker, we advised job modification.

Spirometry is the most significant test for diagnosing COPD. FVC, FEV_1_, and the FEV_1_/FVC ratio are measured by spirometry and are considered to be the most useful variables. Airflow limitation is defined by a FEV_1_/FVC value < 0.7. To rule out asthma and diagnose COPD, post-bronchodilator spirometry should be per formed at least once for patients with airflow limitation [11]. The European Respiratory Society states that acute bronchodilator reversibility is positive when the improvements in FEV_1_ and/or FVC are both > 12 % of the predicted value and exceed 200 ml after a subject uses a bronchodilator [12]. While a slight increase in FEV_1_ is normal after the use of a bronchodilator, a diagnosis of asthma should be made when FEV_1_ increases significantly. Post-bronchodilator spirometry produces measures representing the maximum pulmonary function at the time of testing, serves as a reference for evaluating prognosis, and helps determine that the bronchodilator treatment is effective (if bronchodilator reversibility shows a meaningful increase) [13]. In this study, the patient’s FEV_1_/FVC ratio was 0.46 and the proportion of FEV_1_ predicted was 49 %. Thus, his COPD severity was classified as GOLD 3.

If irreversible airflow limitation progresses, the work ability of individuals with COPD can consistently weaken. Thus, early treatment and improvement of exacerbating factors are crucial in workers with COPD. However, the diagnosis and treatment maintenance of COPD is difficult during the early stages, since the disease is not recognized widely and airflow limitation progresses gradually for an extended period [14]. Patients at an early stage of the disease are either unaware of their condition or are reluctant to consult their physicians concerning their respiratory symptoms [1]. According to a previous study, only 2.4 % of COPD patients are diagnosed, and only 2.1 % receive treatment. Therefore, it is necessary to diagnose and man age early COPD by performing post-bronchodilator spirometry during a secondary screening test, which is intended for individuals whose FEV_1_/FVC score falls below 0.7 on a primary screening test. In addition, studies suggest that annual spirometry screening should be performed for current smokers and ex-smokers > 40 years of age [15, 16].

The symptoms of dyspnea vary greatly among individuals with similar degrees of airflow limitation [17]. Therefore, GOLD has not only devised spirometric classifications of COPD patients, but also classifies patients according to the severity of disease, and takes into consideration breathlessness and risk of exacerbation. The CAT is useful for evaluating life quality since it reflects respiratory symptoms, degree of daily activeness, and self-esteem. The mMRC scale refers to the degree of dyspnea, where a higher score indicates worse prognosis and higher mortality risk [18].

COPD patients experience a decline in exercise capacity due to cardiovascular complications, in addition to problems with voluntary ventilation capacity due to airflow limitation [19]. Therefore, it is very important to measure maximum oxygen uptake in order to prescribe COPD patients appropriate work intensity levels. Methods of evaluating exercise capacity include climbing stairs, the 6-min walk test, the shuttle walk test, and the cardiopulmonary exercise test [20, 21].

The 6-min walk test is used widely in many medical institutions since it is simple and practical. It is performed in a 30 m corridor and requires no specialized equipment or professional testers. Kim et al. presented the following prediction equation, which calculates maximum oxygen uptake in terms of the distance walked in 6 min and the carbon monoxide diffusing capacity of the lungs: (DL_CO_) = (274.306 ×  FEV_1_) + (36.242 × DL_CO_) + (0.007 × 6-minwalk distance × body weight) – 84.867 [22]. The 6-min walk test differs from the exercise stress test because it is executed under a sub-maximum degree of exercise, while the exercise stress test is based on maximum exercise-load [23]. However, as Kim et al. reported, the 6-min walk test can be used as an alternative when performance of the exercise stress test is impossible [22]. The 6-min walk test is mutually complementary to the exercise stress test and should be more actively introduced as it better reflects daily activity.

The exercise stress test is useful for quantifying exercise capacity by measuring VO_2max_ [6, 24]. Additionally, it provides information on exercise limitations caused by COPD. The VO_2max_ value calculated from the exercise stress test can be used as an objective basis for work task and work duration adjustments [25]. The most widely used exercise stress test is the Bruce treadmill test, which is designed to diagnose coronary artery disease and to assess functional capacity [26]. An individual begins the test by walking on a treadmill inclined at 10 % at a speed of 2.74 km/h. The test comprises ten levels, with slope and speed increasing every 3 min at each level. The overall score is based on the duration of the test. The high exercise intensity and relatively long intervals are likely to be a heavy burden on patients with moderate to severe pulmonary disease. For these patients, a modified Bruce protocol, which is a predictive submaximal exercise test, may be useful [27]. Atrophy of skeletal muscle caused by severe COPD may prevent patients from performing the exercise stress test [28].

Contraindications for the exercise stress test should be assessed prior to its commencement, and the test methods and purpose should be explained clearly to the patient. It is important to acknowledge the risks of the test and take notice of termination criteria such as dizziness, severe fatigue , dyspnea, or chest pain. The reliability of the test, which is directly associated with the patient’s effort, can be determined if the heart rate of the patient reaches at least 85 % of the maximum predicted heart rate.

According to the Bruce protocol, the total duration of the test can be converted to a VO_2max_ (ml/kg/min) value using the following equation (in which ‘T’ is the total time on the treadmill, measured as a fraction of a minute ): 14.76 - (1.379 × T) + (0.451 × T^2^) - (0.012 × T^3^) [29]. VO_2max_ refers to the maximum amount of oxygen an individual uses per minute during dynamic exercise. In this study, the patient’s VO_2max_ was 19.16 ml/kg/min, which is considered ‘very poor’ according to his age group distribution [8].

A previous study suggested that the cut-offs for acceptable workload should be as follows (based on the percentage of VO_2max_ per physical work period): 28.5 % for 12 h, 31 % for 10 h, 34 % for 8 h, and 43.5 % for 4 h [10]. Thus, our patient was advised to work only 8 h when his work tasks required a VO_2max_ value < 34 % (≤6.51 ml/kg/min). Therefore, if the patient’s oxygen demand during 8full-time work hours exceeded 34 % of his VO_2max_, his job specifications had to be modified to allow for a reduction of work hours or work aerobic demand.

When calculating the aerobic demand of work, the total hours worked, tasks performed, work intensity of each task, and number of hours spent on each task must be considered. Approximating aerobic demand for specific tasks can be achieved by using the reference data from Ainsworth et al. [9]. Based on these values, field supervision was deemed inappropriate for our patient, and he was advised to continue working on his previous, sedentary tasks.

Although the exercise stress test was stopped due to abnormal elevation of systolic pressure, the patient complained of dyspnea as he entered stage 4, and his heart rate didn’t increase in stage 4 after reaching its maximum in stage 3. This indicates that the test result adequately reflects the patient’s maximum cardiopulmonary capacity. The maximum oxygen uptake (19.09 mL/kg/min) calculated in terms of the maximum heart rate from the actual exercise stress test and the resting heart rate from Uth–Sørensen–Overgaard–Pedersen estimation was similar to the maximum oxygen uptake (19.16 mL/kg/min) calculated in terms of the time that the exercise stress test continued in this case [30, 31]. However, it is assumed that proper control of hypertension could help getting a more precise maximum oxygen uptake (VO_2max_), which is a limitation of this study.

Those affected with COPD should be aware of workplace exposure to noxious particles and gases, and should wear respiratory protective equipment (RPE). However, inappropriate use of RPE can result in carbon dioxide rebreathing , even under conditions of low work intensity, such as talking with colleagues [32]. Gas exchange abnormalities due to carbon dioxide rebreathing can cause hypoxemia or hypercapnia in stable COPD patients with severe airflow obstruction, and can impede oxygen supply and aggravate COPD [33]. PAPR are air-fed respirators that supply filtered air and are equipped with a visor [34]. PAPR are appropriate for workers with COPD as they provide a continuous supply of oxygen, even in enclosed spaces with reduced oxygen levels. Furthermore, these devices do not increase work burden, as they are lightweight, confer high mobility, and can be removed easily.

Early diagnosis of COPD and assessment of fitness to work are becoming increasingly important commensurate with the expected increase in COPD incidence in Korea. However, the current guidelines and tests used for the diagnosis and fitness to work assessment of COPD patients in Korea are inadequate. First, it is challenging to correctly diagnose COPD with a resting lung function test performed without a bronchodilator. Second, disease severity is difficult to evaluate, as there are no empirical assessment scales available to examine symptoms in patients with abnormal spirometry results. Finally, the exercise stress test is required to determine loss of work ability.

Overall, as this case study demonstrates, calculating VO_2max_ from exercise stress test results is beneficial to assess work ability. Since there are various factors affecting the exercise capacity of individuals with COPD, differences can arise between exercise capacity level and the degree of pulmonary dysfunction calculated by spirometry [35, 36]. Therefore, individuals with COPD who experience fatigue and dyspnea in their work environment subsequent to workload modifications should undergo an exercise stress test to thoroughly gauge their exercise capacity level.

## Consent

We performed a retrospective chart review and collected no personal identification data. The Institutional Review Board of Inje University Haeundae Paik Hospital approved our waiver of written informed consent and review exemption (IRB No. 129792-2014-120).
